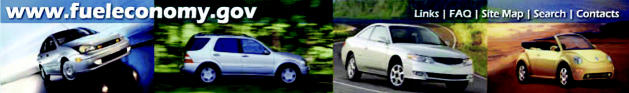# EHPnet: www.fueleconomy.gov

**Published:** 2005-04

**Authors:** Erin E. Dooley

The 2005 North American International Auto Show in Detroit saw the introduction of a number of highly efficient cars, including several new gas–electric hybrid models. The market is expected to see even more hybrid and other alternative models in the future, with U.S. sales of hybrids alone projected to grow from 80,000 in 2004 to more than 400,000 in 2008, according to marketing information firm J.D. Power and Associates. Now the U.S. Department of Energy and Environmental Protection Agency have teamed up to develop a website, **http://www.fueleconomy.gov/**, where consumers can learn more about new automobile technologies and get help in selecting a fuel-efficient vehicle from the models currently on the market.

The site’s Why Is Fuel Economy Important? section answers its titular question with information contained in four subsections: Protect the Environment, Reduce Oil Imports, Conserve Resources for Future Generations, and Save Money. The Protect the Environment subsection describes how petroleum extraction and use contributes to global warming, oil spills, and air pollution. Currently 133 million Americans live in areas that fail at least one National Ambient Air Quality Standard, and vehicles produce 25–75% of the chemicals that pollute the air. All new vehicles must meet federal emissions standards, and those that use fuel more efficiently may produce less pollution over time than those with lower fuel economy.

The online *Model Year 2005 Fuel Economy Guide*, which can be downloaded for free, offers a list of the year’s best-in-category automobiles as well as a complete categorical listing of the year’s models sold in the United States and fuel economy figures for each. The listings include hybrid and other alternative fuel vehicles. By selecting the Find and Compare Cars link, consumers can search an online database by model year, class, make, miles per gallon, and fuel requirements to find a vehicle to meet their personal requirements.

For people looking to learn more about how hybrid and other alternative vehicles work, the Advanced Technology section provides short descriptions of systems and materials incorporated into vehicles to help improve their fuel economy. These include aerodynamic design and lightweight materials such as aluminum, plastics, magnesium, carbon fiber, and metal matrix composites. This section also offers links to outside sites that provide more in-depth information on these technologies.

Looking for cheap gas? Want to know how to improve your fuel economy? The site answers these two questions with separate sections. The Gasoline Prices section provides price information at the local, state, regional, and national level, and allows visitors to find the cheapest gas nearby. The Gas Mileage Tips section provides information on driving more efficiently, keeping cars in shape, planning and combining trips, and choosing more efficient vehicles.

The Answers to Your Questions link on the homepage leads to information on how greenhouse gas emissions are determined, whether to use premium or regular gasoline, how fuel economy estimates are obtained, the gas guzzler tax, and tax incentives for alternative fuel and hybrid vehicles. There is also a Links page that routes visitors to online car buyer guides, electronic magazines and news sites, safety and environmental research sites, advanced technology information, and manufacturer home-pages.

## Figures and Tables

**Figure:**